# Reliability and validity of the Croatian version of Consultation and Relational Empathy (CARE) Measure in primary care setting

**DOI:** 10.3325/cmj.2015.56.50

**Published:** 2015-02

**Authors:** Miroslav Hanževački, Trpimir Jakovina, Žarko Bajić, Aran Tomac, Stewart Mercer

**Affiliations:** 1 Zagreb Zapad Health Care Center, Zagreb, Croatia; 2 Department of Psychological Medicine, Zagreb University Hospital Center, Zagreb, Croatia; 3 Biometrika Healthcare Research, Zagreb, Croatia; 4 Department of Primary Care, University of Glasgow, Glasgow, UK

## Abstract

**Aim:**

To translate the Consultation and Relational Empathy (CARE) Measure into Croatian and validate the Croatian version of the questionnaire.

**Methods:**

A cross-sectional study was conducted in July 2011 in 8 general practices (GP) in Croatia. Following two stages of translation, back-translation, and pilot testing, the Croatian version of the CARE was tested on 568 consecutive patients.

**Results:**

Face validity was high, the number of missing values was low (9%), and the internal consistency (Cronbach’s alpha) was 0.77. A principal component analysis of 10 CARE Measure items extracted two components with eigenvalues >1. These two components explained 43.6% of the total instrument variance.

**Conclusion:**

The Croatian version of the CARE Measure had acceptable reliability and face validity, but its intended component structure was not reproduced and further research is needed to understand its dimensionality.

Physician empathy is widely regarded as an essential component of primary health care consultations and is central to the physician-patient relationship (1,2). In the clinical context, it is usually defined as the physician’s ability to understand the patient's situation, perspective, and feelings; to communicate that understanding to the patient, check its accuracy, and to act upon it in a helpful therapeutic way (3). Empathy has been linked to a number of benefits in health care encounters including patient satisfaction, patient enablement, and better health outcomes (3-5). It may have both immediate and long-term effects on the patient (6). Attempts to measure empathy from a neurobiological perspective, although promising, will not be applicable in health care consultation settings in the near future. Several psychometric tools have been developed to measure physician’s empathy, with the Jefferson Scale of Empathy being the most referenced one (7). However, none of these scales have been designed specifically for the primary care setting and the majority of them is administered by physicians rather than self-administered (8-10). This is the reason why we chose the Consultation and Relational Empathy (CARE) Measure, which is widely used for the patient-rated assessment of physician empathy in the primary health care setting and which requires only 5-10 minutes to complete (11,12). Like many other physician empathy scales, the CARE measures situational empathy and ignores dispositional empathy, which is understood as physician’s character trait. The CARE measure has been validated in English, German, and Chinese (4,11,12). The aim of this study was to translate the questionnaire into Croatian and validate the Croatian version, determining its face validity, reliability, and dimensionality.

## Methods

### Data collection

We conducted a cross-sectional study on a consecutive sample of patients within a convenient sample of 8 GP in urban areas of Zagreb and Split, using the Croatian translation of the original (English) CARE Measure (Supplementary material(web extra material 1)).

### Translation of the CARE Measure

The CARE Measure has 10 items with response options ranging from poor to excellent (scoring 1-5) and “not applicable” option. The final score ranges from 10 to 50. The questionnaire was translated in two stages. In the first stage, the original English CARE questionnaire was translated into Croatian by two independent translators, who had no prior knowledge of the questionnaire. After both translations were completed, the two translators compared their translations and jointly produced a third translation. The harmonized translation was then given to a native English translator to back-translate it into English. After the back-translation and the original author’s assessment and agreement, this draft Croatian version was piloted on 40 patients in 2 GP offices (23 + 17 patients), and tested further on consecutive patients in 7 GP offices. In this translation stage, answers provided by 505 patients were collected. Principal component analysis resulted in three components. Since the observed results were not in concordance with publications in the UK, Germany, and Hong Kong, where the CARE was a one-dimensional scale, we had to re-evaluate the whole process. Even though all of the steps in translation/back-translation were performed adequately we agreed that the small differences in meaning between the original and Croatian version most likely led to differences in scale dimensionality. Consequently, we agreed to translate the original English questionnaire once again. In the second translation stage, we had the original English questionnaire translated by two independent translators with considerable experience in medical translations, who had not participated in the first translation. This version was shown to 20 consecutive patients in 2 GP practices to check its face validity (6 male and 14 female patients, aged 29 to 79, with various comorbidities, educational levels, and household incomes) and was used among 568 consecutive patients in 8 GP practices from 1 to 7 June 2011.

The GPs asked consecutive patients to participate until each recruited at least 60 patients. After having gained informed consent, the GPs explained the aim of the research and how to fill in the questionnaire. Nurses measured the duration of each visit and, for the patients who accepted to participate in the study, the time necessary to complete the questionnaire. The GPs were asked to clearly state that the questionnaire was anonymous and the answers would not influence their relationship with the physician in any way. The presence of any co-morbidity and patients’ self-assessed income status was recorded (average, below average, or above average). Additionally, patients were asked how long they had been treated by this GP and if they would recommend him or her to a friend or relative. The patients filled in the questionnaire in the nurse’s office by themselves and dropped it in a non-transparent box. In the second translation stage, we also compared the patients who accepted to participate and those who refused, and compared the percentage of non-applicable results for both translations. Only the data collected in the second translation stage were taken into account when analyzing validity, reliability, and dimensionality. The approval of ethics committees of Health Care Centers in Zagreb and Split were obtained, and all patients provided written informed consent.

### Statistical analysis

Internal validity was determined by Cronbach’s alpha and dimensionality with a principal component analysis. Varimax rotation with Kaiser normalization was used, as well as Kaiser criterion of retaining components with eigenvalues higher than 1. Normality of distribution was tested by Kolmogorov-Smirnov test, and median and interquartile range were used as measures of central tendency and variability. The level of significance was set to 5% (*P* < 0.05). All analyses were carried out using SPSS 17.0 (SPSS Inc., Chicago, IL, USA).

## Results

947 participants were recruited in 8 GP practices and 568 (60%) agreed to participate in the validation process. The patients who agreed to participate and those who did not, as well as the patients from 8 GPs were comparable in respect to age, sex, and presence of chronic illnesses, except patients from one GP (GP 5), who all had chronic diseases (Table 1). The average visit duration was 6.8 minutes and 50% lasted between 6-10 minutes (Table 2). All patients were examined by their regular GP.

**Table 1:** Characteristics of participants and non-participants from 8 general practices (GP)

			Zagreb (n = 443)												Split (n = 125)				Patients refused to participate (n = 379)	
n (%)	All patients (n = 568)		GP 1 (n = 75)		GP 2 (n = 74)		GP 3 (n = 59)		GP 4 (n = 77)		GP 5 (n = 70)		GP 6 (n = 88)		GP 7 (n = 64)		GP 8 (n = 61)	
Age*	53	(41-62)	49	(41-67)	53	(43-61)	54	(42-62)	54	(40-67)	53	(39-61)	50	(38-61)	54	(43-61)	51	(40-60)	53	(41-61)
Sex																				
male	235	(41.4)	26	(34.7)	32	(43.2)	26	(44.1)	33	(42.9)	31	(44.3)	36	(40.9)	26	(40.6)	25	(41.0)	176	(46.4)
female	333	(58.6)	49	(65.3)	42	(56.8)	33	(55.9)	44	(57.1)	39	(55.7)	52	(59.1)	38	(59.4)	36	(59.0)	203	(53.6)
Chronic illness																				
yes	361	(66.5)	46	(61.3)	41	(55.4)	40	(67.8)	50	(64.9)	45	(100.0)	58	(65.9)	40	(62.5)	41	(67.2)	148	(48.4)
no	182	(33.5)	29	(38.7)	33	(44.6)	19	(32.2)	27	(35.1)	0	(0.0)	30	(34.1)	24	(37.5)	20	(32.8)	158	(51.6)
Self-assessed economic status^†^																				
above the average	104	(18.4)	18	(24.0)	15	(20.3)	6	(10.2)	16	(20.8)	13	(18.6)	17	(19.3)	8	(12.5)	11	(18.6)		
average or bellow	462	(81.6)	57	(76.0)	59	(79.7)	53	(89.8)	61	(79.2)	57	(81.4)	71	(80.7)	56	(87.5)	48	(81.4)		

*Median (interquartile range).

^†^Data were not collected for patients who refused to participate.

**Table 2:** Consultation characteristics

Characteristic	n (%)
Visit duration	
<3 min	65 (11.5)
4-5 min	138 (24.3)
6-10 min	283 (49.9)
>10 min	81 (14.3)
total	567 (100.0)
How long have you been treated by this general practitioner?	
<3 months	19 (3.3)
3-6 months	113 (19.9)
>6 months	436 (76.8)
total	568 (100.0)
How well do you know this general practitioner?	
not well	9 (1.6)
neutral	178 (31.4)
well	276 (48.8)
very well	103 (18.2)
total	566 (100.0)
Would you recommend this general practitioner to a friend/relative?	
no	43 (7.7)
yes	515 (92.3)
total	558 (100.0)

### Details of Croatian CARE Measure

**Figure 1:**
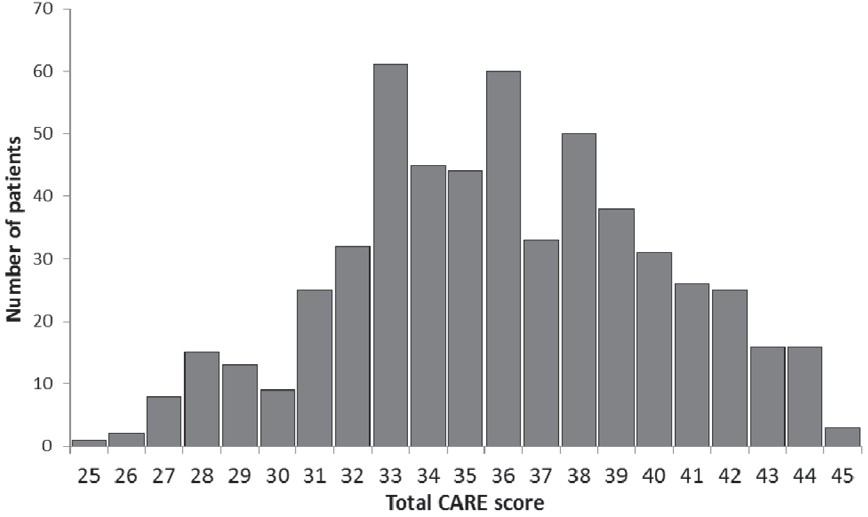
Total Consultation and Relational Empathy (CARE) Measure results distribution

The median CARE Measure score was 36 (IQR, 33-39), ranging from the minimum of 23 to the maximum of 45 (Figure 1). The participants showed a tendency to evaluate their GPs on the individual items as “good” or “very good” (Table 3). 86.9% of all answers to all items were “good” or “very good”.

**Table 3:** Croatian Consultation and Relational Empathy (CARE) Measure particular items distributions

Item, n (%)	Poor		Fair		Good		Very good		Excellent		Total	
Average for all items	5	(0.8)	32	(5.6)	190	(33.6)	302	(53.3)	38	(6.8)	567	(100.0)
1. Making you feel at ease	13	(2.3)	46	(8.1)	205	(36.2)	270	(47.6)	33	(5.8)	567	(100.0)
2. Letting you tell your story	9	(1.6)	24	(4.2)	202	(35.6)	280	(49.3)	53	(9.3)	568	(100.0)
3. Really listening	5	(0.9)	38	(6.7)	181	(32.0)	290	(51.2)	52	(9.2)	566	(100.0)
4. Being interested in you as a whole person	5	(0.9)	36	(6.4)	156	(27.7)	317	(56.2)	50	(8.9)	564	(100.0)
5. Fully understanding your concerns	0	(0.0)	24	(4.2)	208	(36.7)	308	(54.3)	27	(4.8)	567	(100.0)
6. Showing care and compassion	0	(0.0)	21	(3.7)	187	(33.0)	324	(57.1)	35	(6.2)	567	(100.0)
7. Being positive	4	(0.7)	15	(2.6)	169	(29.8)	334	(58.9)	45	(7.9)	567	(100.0)
8. Explaining things clearly	4	(0.7)	42	(7.4)	182	(32.0)	306	(53.9)	34	(6.0)	568	(100.0)
9. Helping you to take control	5	(0.9)	31	(5.5)	216	(38.0)	284	(50.0)	32	(5.6)	568	(100.0)
10. Making a plan of action with you	2	(0.4)	39	(6.9)	197	(34.7)	308	(54.2)	22	(3.9)	568	(100.0)

### Face validity

9% of the answers were not valid and 7% where ticked as non-applicable, with no significant difference among the items. The majority of patients (13 out of 20) could read and fill in the questionnaire by themselves and had no difficulty understanding it. However, 7 patients required minor assistance during answering. These were older patients, who were highly educated and very persistent in discussing the semantic structure of the sentences. However, their remarks were not related to the meaning of the questions. All this indicates satisfactory face validity.

### Construct validity and principal component analysis

Construct validity was evaluated by a principal components analysis (Table 4). The Kaiser-Meyer-Olkin statistical criteria (KMO = 0.814) and the Bartlett Test of sphericity (value 958.58, *P* < 0.001) indicated that the raw data were suitable for the principal component analysis. The principal components analysis extracted two components with eigenvalues higher than 1 (Table 5). The first component explained 24.6% of the variance and the second 19.0%. Both components explained 43.6% of the total variance. The items highly saturated with the first component were related to more concrete situations, while the items highly saturated with the second component were related to more abstract situations. The first principal component (items: 1,2,3,7,8,9,10) had acceptable internal consistency, with Cronbach’s alpha of 0.72. Item total correlations ranged from 0.34 to 0.50. The second subscale (claims 4, 5, 6) did not have acceptable consistency (Cronbach’s alpha = 0.63), with item total correlations ranging from 0.36 to 0.42. For both dimensions, removal of some the items did not increase the Cronbach’s alpha.

**Table 4:** Reliability and homogeneity of the Croatian Consultation and Relational Empathy (CARE) Measure*

Particular items	Scale mean if item deleted	Corrected item-total correlation	Cronbach's alpha if item deleted
1. Making you feel at ease	32.50	0.427	0.751
2. Letting you tell your story	32.35	0.483	0.743
3. Really listening	32.35	0.432	0.75
4. Being interested in you as a whole person	32.30	0.489	0.742
5. Fully understanding your concerns	32.37	0.419	0.752
6. Showing care and compassion	32.30	0.368	0.757
7. Being positive	32.25	0.401	0.754
8. Explaining things clearly	32.42	0.487	0.743
9. Helping you to take control	32.43	0.438	0.749
10. Making a plan of action with you	32.42	0.379	0.757

*Entire instrument: Cronbach’s alpha, 0.77.

**Table 5:** Principal components analysis, structure matrix of the Croatian Consultation and Relational Empathy (CARE) Measure

	Components	
	1st	2nd
(2) Letting you tell your story	0.70	0.13
(1) Making you feel at ease	0.65	0.08
(7) Being positive	0.62	0.08
(3) Really listening	0.59	0.15
(9) Helping you to take control	0.55	0.23
(8) Explaining things clearly	0.54	0.31
(10) Making a plan of action with you	0.39	0.30
(4) Being interested in you as a whole person	0.19	0.79
(5) Fully understanding your concerns	0.11	0.78
(6) Showing care and compassion	0.19	0.61

Participants were divided into subgroups according to the following criteria: city, chronic illnesses, sex, age, and randomly to verify the stability of the two extracted principal components. Randomization was done by SPSS Select cases: Random Sample command with user-specified exact number of cases. SPSS generates a random sample without replacement. Only one principal component was extracted in Zagreb and three in Split. The component extracted in Zagreb explained 32.7% of the variance. The three components extracted in Split explained 61.5% of the variance. When the sample was divided by chronic illnesses, sex, and randomly, the two originally extracted dimensions were preserved.

## Discussion

We translated the English version of the CARE measure to Croatian and tested it among routine patients in public primary health care practices in Croatia. The physicians’ and patients’ comments suggested acceptable face validity. The majority of patients accepted the questionnaire and filled it out without significant problems. Two principal components were extracted and reliability indicated by Cronbach’s alpha was acceptable.

Similar patients’ experiences were found in the United Kingdom and Hong Kong and the number of patients who marked particular items as non-applicable was comparable (4,11,13). The main difference between the Croatian and English version of the CARE Measure was found in dimensionality. We cannot explain the reason for this difference, especially since in the second translation stage, we went to great lengths to preserve the precise meaning of the English terms. Like in the original version, all items were additionally explained in the parentheses. For instance, the item “really listening” was presented in the form: “He/she really listened to me (paying full attention to what I was saying, not looking at his/her notes or computer while I was speaking).” It is possible that the additional explanations for the first subscale items were more concrete, while the second subscale items had more abstract explanations (eg, “not treating me like just a number or connected with me at a human level”). This type of formulation may be more open to interpretation and cannot be unambiguously described as something that did or did not occur. However, this is only a hypothesis that remains to be confirmed by further investigation. Construct validity was supported by positive correlations of both subscales with patients’ willingness to recommend their doctor to a friend or a relative and with recorded consultation duration. Similar findings were found in both the Chinese and English validation (4,9,12). Cronbach’s alpha of the total Croatian CARE measure indicated a satisfactory internal consistency, though again, this was lower than in the English or Chinese versions (4,9,14). Visit duration significantly correlated with the CARE score, which is understandable, as those physicians who are authentically interested in the patient’s perspective are more likely to be perceived as “empathic” and spend more time with the patient (13).

The study has several limitations. First of all, the sample of physicians was not representative of the population of GPs in the country. The questionnaire administration by the GP may also have introduced bias, since the usual practice is that the questionnaire is administered by reception or research staff (15). We chose this form of administration on purpose, as we think that it might become regular practice in the future. Although we consider that the Croatian version of the CARE has acceptable reliability for assessing physician empathy, differences in dimensionality from other versions of the CARE Measure require further examination. These differences may have been caused by the weaknesses of our systematic, non-random sample. Future studies should consider generating a list of all adult patients who have visited the GP at least twice over the past year, then randomly selecting the needed sample size from the list, and invite the participants. This way the sample will be more representative of the targeted population.

It is noteworthy that the majority of patients rated their doctor as good or very good, which may be explained by Croatian patients’ good opinion of GPs’ empathy and care in general (16). Our patients were older than those in the Chinese study, so the high ratings might be explained by their lower expectations from physicians (14,17). We believe that future studies should elucidate the influence of patient’s expectations on reported satisfaction with physician empathy. It is also possible that the high rankings were a consequence of the administration of the questionnaire by the GPs themselves (18,19). Also, most of the studies addressing patient satisfaction with medical care report high patient satisfaction with various elements of care, and the same has been observed for the CARE Measure (19,20).

Despite these limitations**,** we showed the importance of a structured and precise translation process to get a version that exactly matches the purpose of the original self-administered questionnaire. The same translation procedure as well as adequate sampling should be applied in other countries and for other instruments. The lower percentage of non-applicable responses proved to be a very useful parameter, especially in combination with the rate of non-valid answers, which indicated the need for more adequate translation. It is also important that researchers during validation pay attention to patients’ comments and evaluate them thoroughly. This research provides an instrument of acceptable reliability for physician empathy assessment, which is important in the daily care of patients in Croatia and for comparison with other countries where the CARE Measure is validated. However, further research is needed to understand its dimensionality.

## References

[1] Reynolds WJ, Scott B (1999). Empathy. A crucial component of the helping relationship.. J Psychiatr Ment Health Nurs.

[2] Mercer SW, Reynolds WJ (2002). Empathy and quality of care.. Br J Gen Pract.

[3] Derksen F, Bensing J, Lagro-Janssen A (2013). Effectiveness of empathy in general practice: A systematic review.. Br J Gen Pract.

[4] Mercer SW, Jani BD, Maxwell M, Wong S, Watt GC (2012). Patient enablement requires physician empathy: A cross-sectional study of general practice consultations in areas of high and low socioeconomic deprivation in Scotland.. BMC Fam Pract.

[5] Jani BD, Blane DN, Mercer SW (2012). The role of empathy in therapy and the physician-patient relationship.. Research in Complementary Medicine..

[6] Neumann M, Bensing J, Mercer S, Ernstmann N, Ommen O, Pfaff H (2009). Analyzing the “nature” and “specific effectiveness” of clinical empathy: A theoretical overview and contribution towards a theory-based research agenda.. Patient Educ Couns.

[7] Riess H (2010). Empathy in medicine – a neurological perspective.. JAMA.

[8] Katić M, Juresa V, Oresković S (2004). Family medicine in Croatia: past, present, and forthcoming challenges.. Croat Med J.

[9] Hebrang A, Henigsberg N, Erdeljić V, Foro S, Vidjak V, Grga A (2003). Privatization in the health care system of Croatia: Effects on general practice accessibility.. Health Policy Plan.

[10] Davidsen AS (2009). How does the general practitioner understand the patient? A qualitative study about psychological interventions in general practice.. Psychol Psychother.

[11] Mercer SW, Maxwell M, Heaney D, Watt GC (2004). The consultation and relational empathy (CARE) measure: Development and preliminary validation and reliability of an empathy-based consultation process measure.. Fam Pract.

[12] Mercer SW, McConnachie A, Maxwell M, Heaney D, Watt GC (2005). Relevance and practical use of the Consultation and Relational Empathy (CARE) Measure in general practice.. Fam Pract.

[13] Geraghty EM, Franks P, Kravitz RL (2007). Primary Care visit length, quality and satisfaction for standardized patients with depression.. J Gen Intern Med.

[14] Fung CS, Hua A, Tam L, Mercer SW (2009). Reliability and validity of the Chinese version of the CARE measure in a primary care setting in Hong Kong.. Fam Pract.

[15] Kelm Z, Womer J, Walter JK, Feudtner C (2014). Interventions to cultivate physicians empathy: a systematic review.. BMC Med Educ.

[16] Public opinion survey on health care system and Croatian Health Insurance Fund in 2013 [in Croatian]. Available from: http://cdn.hzzo.hr/wp-content/uploads/2014/03/Izvje%C5%A1taj_HZZO_2013.pdf*.* Accessed: February 24, 2015.

[17] Price S, Mercer S, McPherson H (2006). Practitioner empathy, patient enablement, and health outcomes: a prospective study of acupuncture patients.. Patient Educ Couns.

[18] Fitzpatrick R (1991). Surveys of patient satisfaction: Important general considerations.. BMJ.

[19] McKinley R, Stevenson K, Adams S, Manku-Scott T (2002). Meeting patient expectations of care: the major determinant of satisfaction with out-of-hours primary medical care?. Fam Pract.

[20] Chen JY, Tao M, Tisnado D, Malin J, Ko C, Timmer M (2008). Impact of physician-patient discussions on patient satisfaction.. Med Care.

