# Supplementary material for: Reliability and validity of the Croatian version of Consultation and Relational Empathy (CARE) Measure in primary care setting
Source: Croat Med J. 2015 Feb;56(1):50–6. doi: 10.3325/cmj.2015.56.50 (PMC4364352; doi:10.3325/cmj.2015.56.50)
Supplement: Supplementary material [file CroatMedJ_56_s003.pdf]

# CARE Patient Feedback Measure for

*** Type name of Practitioner here ***

Please write today's date here:

☐☐ / ☐☐ / ☐☐
D D M M Y Y

**Please rate the following statements about today's consultation.**

Please mark the box like this ☑ with a ball point pen. If you change your mind just cross out your old response and make your new choice. Please answer every statement.

| How good was the practitioner at... | Poor | Fair | Good | Very Good | Excellent | Does not apply |
|---|---|---|---|---|---|---|
| **1) Making you feel at ease** (introducing him/herself, explaining his/her position, being friendly and warm towards you, treating you with respect; not cold or abrupt) | ☐ | ☐ | ☐ | ☐ | ☐ | ☐ |
| **2) Letting you tell your "story"** (giving you time to fully describe your condition in your own words; not interrupting, rushing or diverting you) | ☐ | ☐ | ☐ | ☐ | ☐ | ☐ |
| **3) Really listening** (paying close attention to what you were saying; not looking at the notes or computer as you were talking) | ☐ | ☐ | ☐ | ☐ | ☐ | ☐ |
| **4) Being interested in you as a whole person** (asking/knowing relevant details about your life, your situation; not treating you as "just a number") | ☐ | ☐ | ☐ | ☐ | ☐ | ☐ |
| **5) Fully understanding your concerns** (communicating that he/she had accurately understood your concerns and anxieties; not overlooking or dismissing anything ) | ☐ | ☐ | ☐ | ☐ | ☐ | ☐ |
| **6) Showing care and compassion** (seeming genuinely concerned, connecting with you on a human level; not being indifferent or "detached") | ☐ | ☐ | ☐ | ☐ | ☐ | ☐ |
| **7) Being positive** (having a positive approach and a positive attitude; being honest but not negative about your problems) | ☐ | ☐ | ☐ | ☐ | ☐ | ☐ |
| **8) Explaining things clearly** (fully answering your questions; explaining clearly, giving you adequate information; not being vague) | ☐ | ☐ | ☐ | ☐ | ☐ | ☐ |
| **9) Helping you to take control** (exploring with you what you can do to improve you health yourself; encouraging rather than "lecturing" you) | ☐ | ☐ | ☐ | ☐ | ☐ | ☐ |
| **10) Making a plan of action with you** (discussing the options, involving you in decisions as much as you want to be involved; not ignoring your views) | ☐ | ☐ | ☐ | ☐ | ☐ | ☐ |

**Comments:** If you would like to add further comments on this consultation, please do so here.

© CARE SW Mercer, Scottish Executive 2004: The CARE Measure was orginially developed by Dr Stewart Mercer and colleagues as part of a Health Service Research Fellowship funded by the Chief Scientist Office of the Scottish Executive (2000-2003).

**Dob** ___________ **Spol M Ž**

**Je li ovo Vaš stalni liječnik DA NE**
**Koliko ste dugo kod ovog liječnika/ice <3 mjeseca 3-6 mjeseci >6 mjeseci**

---

## Koliko je Vaš liječnik/ica tijekom pregleda uspio u sljedećem:

1. **Osjećao/**la **sam se ugodno** (bio/la je prijateljski raspoložen/a, ponašao/la se s poštovanjem, nije bio/la hladan ili me prekidao/la)
   **loše, prihvatljivo, dobro, jako dobro, izvrsno** Nevažno __

2. **Dozvolio/**la **je da ja 'ispričam priču'** (dajući mi vremena da u potpunosti opišem svoju bolest vlastitim riječima, ne prekidajući me ili preusmjeravajući me)
   **loše, prihvatljivo, dobro, jako dobro, izvrsno** Nevažno__

3. **Zaista je slušao/**la (poklanjajući punu pažnju onome što govorim, ne gledajući u zabilješke ili računalo dok govorim)
   **loše, prihvatljivo, dobro, jako dobro, izvrsno** Nevažno__

4. **Bio/la je zainteresiran/**a **za mene kao potpunu osobu** (pitajući ili poznavajući značajne detalje mog života, situacije, ne tretirajući me 'samo kao broj)
   **loše, prihvatljivo, dobro, jako dobro, izvrsno** Nevažno__

5. **U cijelosti je shvatio/**la **moju zabrinutost** (pokazujući da je razumio/la zabrinutost, ne propuštajući ili zanemarujući ništa)
   **loše, prihvatljivo, dobro, jako dobro, izvrsno** Nevažno__

6. **Pokazao/**la **je brigu i suosjećanje** (izgledao/la je iskreno zainteresiran i povezan samnom na ljudskoj razini, a ne kao da je indiferentan ili odsutan)
   **loše, prihvatljivo, dobro, jako dobro, izvrsno** Nevažno__

7. **Bio/la je pozitivan, optimističan/a** (imao/la je pozitiva stav i pristup, iskren ali ne negativan)
   **loše, prihvatljivo, dobro, jako dobro, izvrsno** Nevažno__

8. **Objasnio je sve vrlo jasno** (u potpunosti odgovorio na moja pitanja, dajući adekvatne informacije, nije bio/la nejasan/a)
   **loše, prihvatljivo, dobro, jako dobro, izvrsno** Nevažno__

9. **Pomogao mi je preuzeti aktivnu ulogu u brizi o vlastitom zdravlju** (istražujući što mogu sam/a učiniti da poboljšam svoje zdravlje, ohrabrujući me bez 'govora s visoka')
   **loše, prihvatljivo, dobro, jako dobro, izvrsno** Nevažno__

10. **Zajedno smo napravili plan 'aktivnosti'** (raspravljajući o različitim mogućnosti, uključio/la me u odluke koliko god je bilo moguće, ne ignorirajući moje stavove)

**loše, prihvatljivo, dobro, jako dobro, izvrsno** **Nevažno__**

Prilog 3.

## PEI upitnik

**Molimo odgovorite na svih šest pitanja (tvrdnji) na način da odgovarajuću kućicu u tablici označite križićem**

### Kao rezultat Vašeg današnjeg posjeta liječniku, osjećate li da ste...

| | Puno bolje | Bolje | Jednako ili lošije |
|---|---|---|---|
| **Sposoban nositi se sa životom** | | | |
| **Sposoban razumijeti svoju bolest** | | | |
| **Sposoban nositi se sa svojom bolešću** | | | |
| **Sposoban održavati sebe zdravim** | | | |

| | Puno više | Više | Jednako ili manje |
|---|---|---|---|
| **Čvrsto uvjeren / siguran u svoje zdravlje** | | | |
| **Sposoban sam sebi pomoći** | | | |

---

**Biste li ovog liječnika preporučili prijateljima ili rodbini?** **DA** **NE**

**Kako ste sveukupno zadovoljni ovim liječnikom**
**Vrlo nezadovoljan nezadovoljan srednje zadovoljan vrlo zadovoljan**

**Kako biste procjenili svoja mjesečna primanja?**
**ispodprosječna prosječna iznadprosječan**

---

**Sestra mjeri duljinu konzultacije: <5min 5-10min >10 min**
